# Fulminant Giant Cell Myocarditis vs. Lymphocytic Myocarditis: A Comparison of Their Clinical Characteristics, Treatments, and Outcomes

**DOI:** 10.3389/fcvm.2021.770549

**Published:** 2021-12-03

**Authors:** Yuxiao Hu, Jie Ren, Xueqi Dong, Di Zhang, Yi Qu, Chunxue Yang, Yang Sun, Jinghui Li, Fang Luo, Wei Wang, Huanhuan Wang, Ping Qing, Shihua Zhao, Jie Huang, Litian Yu, Yaxin Liu, Huiqiong Tan

**Affiliations:** ^1^Emergency and Critical Care Center, National Center for Cardiovascular Diseases of China, Fuwai Hospital, Chinese Academy of Medical Sciences, Peking Union Medical College, Beijing, China; ^2^Department of Cardiovascular Surgery, National Center for Cardiovascular Diseases of China, Fuwai Hospital, Chinese Academy of Medical Sciences, Peking Union Medical College, Beijing, China; ^3^Department of Pathology, National Center for Cardiovascular Diseases of China, Fuwai Hospital, Chinese Academy of Medical Sciences, Peking Union Medical College, Beijing, China; ^4^Magnetic Resonance Center, National Center for Cardiovascular Diseases of China, Fuwai Hospital, Chinese Academy of Medical Sciences, Peking Union Medical College, Beijing, China; ^5^Department of Cardiology, National Center for Cardiovascular Diseases, Fuwai Hospital, Chinese Academy of Medical Sciences, Peking Union Medical College, Beijing, China

**Keywords:** fulminant myocarditis, giant cell myocarditis, lymphocytic myocarditis, myocardial biopsy, heart transplantation, outcome

## Abstract

**Objectives:** Fulminant myocarditis (FM) is a rapidly progressive and frequently fatal form of myocarditis that has been difficult to classify. This study aims to compare the clinical characteristics, treatments and outcomes in patients with fulminant giant cell myocarditis (FGCM) and fulminant lymphocytic myocarditis (FLM).

**Methods and Results:** In our retrospective study, nine patients with FGCM (mean age 47.9 ± 7.5 years, six female) and 7 FLM (mean age 42.1 ± 12.3 years, four female) patients confirmed by histology in the last 11 years were included. Most patients with FGCM and FLM were NYHA functional class IV (56 vs. 100%, *p* = 0.132). Patients with FGCM had significantly lower levels of high-sensitivity C-reactive protein [hs-CRP, 4.4 (2.0–10.2) mg/L vs. 13.6 (12.6–14.6) mg/L, *P* = 0.004, data shown as the median with IQR], creatine kinase-myoglobin [CK-MB, 1.4 (1.0–3.2) ng/ml vs. 14.6 (3.0–64.9) ng/ml, *P* = 0.025, median with IQR], and alanine aminotransferase [ALT, 38.0 (25.0–61.5) IU/L vs. 997.0 (50.0–3,080.0) IU/L, *P* = 0.030, median with IQR] and greater right ventricular end-diastolic diameter (RVEDD) [2.9 ± 0.3 cm vs. 2.4 ± 0.6 cm, *P* = 0.034, mean ± SD] than those with FLM. No differences were observed in the use of intra-aortic balloon pump (44 vs. 43%, *p* = 1.000) and extracorporeal membrane oxygenation (11 vs. 43%, *p* = 0.262) between the two groups. The long-term survival rate was significantly lower in FGCM group compared with FLM group (0 vs. 71.4%, *p* = 0.022). A multivariate cox regression analysis showed the level of hs-CRP (hazard ratio = 0.871, 95% confidence interval: 0.761–0.996, *P* = 0.043) was an independent prognostic factor for FM patients. Furthermore, the level of hs-CRP had a good ability to discriminate between patients with FGCM and FLM (AUC = 0.94, 95% confidence interval: 0.4213–0.9964).

**Conclusions:** The inflammatory response and myocardial damage in the patients with FGCM were milder than those with FLM. Patients with FGCM had distinctly poorer prognoses compared with those with FLM. Our results suggest that hs-CRP could be a promising prognostic biomarker and a hs-CRP level of 11.71 mg/L is an appropriate cutoff point for the differentiating diagnosis between patients with FGCM and FLM.

## Introduction

Fulminant myocarditis (FM) is the most severe form of acute myocarditis characterized by a progressively rapid decline in cardiac function, which often requires inotropes and/or mechanical circulation support (1). The in-hospital mortality rate of FM is as high as 40% despite the comprehensive treatments (2, 3). Histological confirmation of myocarditis by endomyocardial biopsy (EMB) is the reference standard for the diagnosis of FM (4). Additionally, EMB is essential to differentiate specific histological subtypes like giant cell myocarditis from lymphocytic myocarditis (5). Despite being highly recommended in patients with fulminant myocarditis by recent scientific statements (6, 7), EMB is often considered invasive and rarely performed.

Giant cell myocarditis (GCM) is a rare and frequently fatal form of myocarditis (8, 9). It manifests a great variety of clinical courses, often presents with progressive heart failure, and sometimes presents with ventricular tachycardia (10–12). Lymphocytic myocarditis is the most common form of myocarditis (5). Viral symptoms often precede the appearance of cardiac symptoms (13–16). There are some differences in the etiology, clinical manifestations, treatments and prognoses between GCM and lymphocytic myocarditis. GCM is considered to be primarily autoimmune because of its association with numerous autoimmune disorders (12), thymoma (17), and drug hypersensitivity (18). Early immunosuppressive therapy is recommended for patients with GCM, while the role of immunosuppressive agents in lymphocytic myocarditis remains controversial (5, 19). A recent report on 163 FM patients confirmed by EMB demonstrated that patients with GCM have significantly worse prognoses compared with lymphocytic myocarditis (20).

However, few reports provide a comprehensive comparison of differences between fulminant giant cell myocarditis (FGCM) and fulminant lymphocytic myocarditis (FLM), particularly in Asian populations. Here we describe the differences in clinical manifestations, laboratory findings, echocardiographic features, treatments, and outcomes of patients with histologically proven myocarditis. Furthermore, biomarkers associated with differential diagnosis and prognosis evaluation in FM patients are discussed in this study.

## Methods

### Study Population

From January 2010 to 2021 May, a total of 16 patients with histologically diagnosed as GCM and lymphocytic myocarditis at Fuwai Hospital were enrolled in this study. Myocardial samples were obtained from the explanted hearts or endomyocardial biopsies. The pathologic criteria for GCM are a diffuse or multifocal inflammatory infiltrate consisting of lymphocytes with multinucleated giant cells associated with myocyte damage (21). Characteristic histopathology of lymphocytic myocarditis is an exclusively or predominately lymphocytic infiltrate with inconspicuous numbers of plasma cells, macrophages, and/or neutrophils (22). The diagnosis of FM was based on previously published reports (6, 19, 23), as a low cardiac output syndrome requiring inotropes and/or MCS.

### Data Collection

The following information of each patient were collected from medical records: demographics (age, sex, and body mass index), comorbidities, main clinical manifestations, results of admission laboratory tests and echocardiography, and the details of treatments with drugs and devices. The endpoint of this study was death or cardiac transplantation. The study followed the tenets of the Declaration of Helsinki and was approved by the Institutional Ethical Committee. All participating patients signed informed consent.

### Statistical Analysis

Categorical variables were expressed as percentages and continuous variables were expressed mean ± SD or median (IQR) as appropriate. Categorical variables were analyzed by chi-square tests or Fisher's exact test. For comparing continuous variables, the student's *t*-test or Mann–Whitney *U*-test was used as appropriate. Kaplan-Meier (K–M) analysis was performed to compare the survival differences of the patients with FLM and FGCM, and the significance of the difference between the two groups was examined by the log-rank test. The relationships between variables and mortality were assessed by the Cox proportional hazards regression analysis. The receiver operating characteristic (ROC) curve was used to assess the accuracy of predictions. A two-sided *P* < 0.05 was considered significant. All statistical analysis was performed using IBM SPSS Statistics software version 23.0 (IBM Corp., Armonk, NY, USA).

## Results

### Demographic Data and Clinical Manifestations

**Figure 1:**
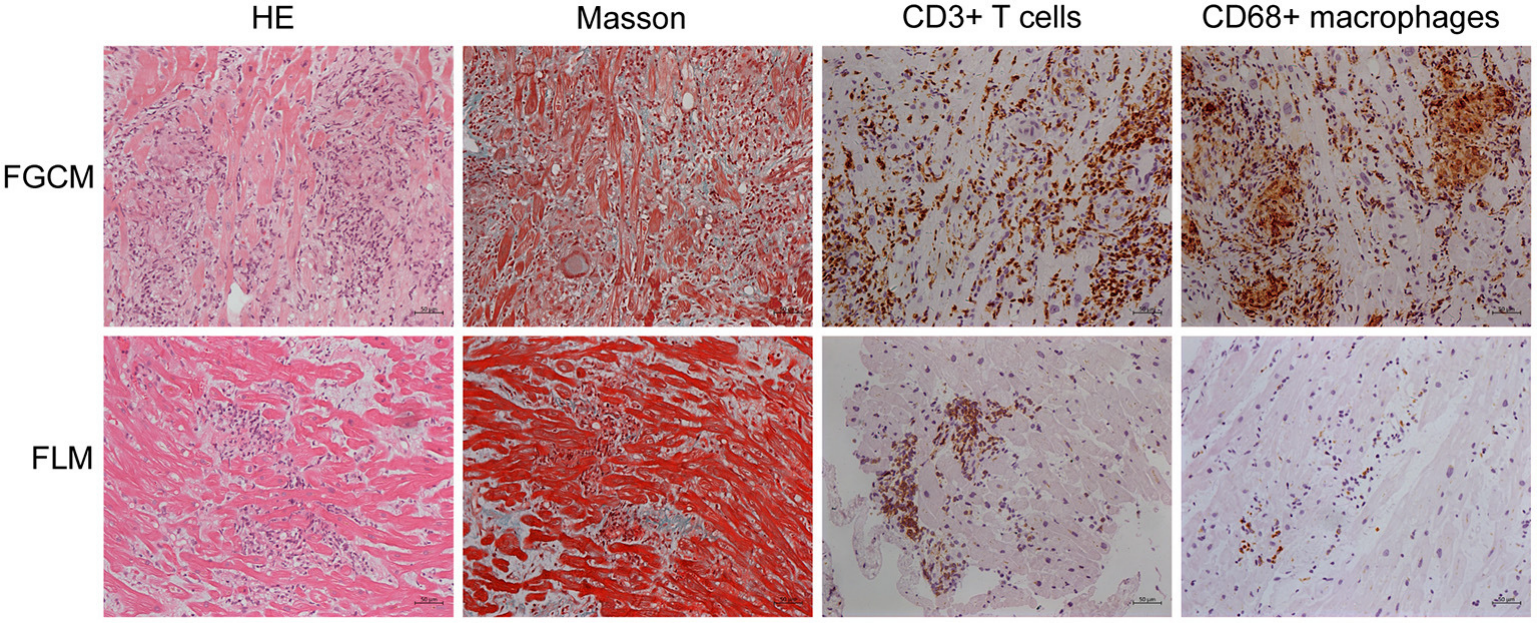
Representative histopathology and immunopathology of patients with FGCM (upper line) and FLM (lower line). First column, hematoxylin and eosin (H&E); second column, Masson trichrome staining; third column, staining with anti-CD3 antibody; fourth column, staining with anti-CD68 antibody.

**Figure 2:**
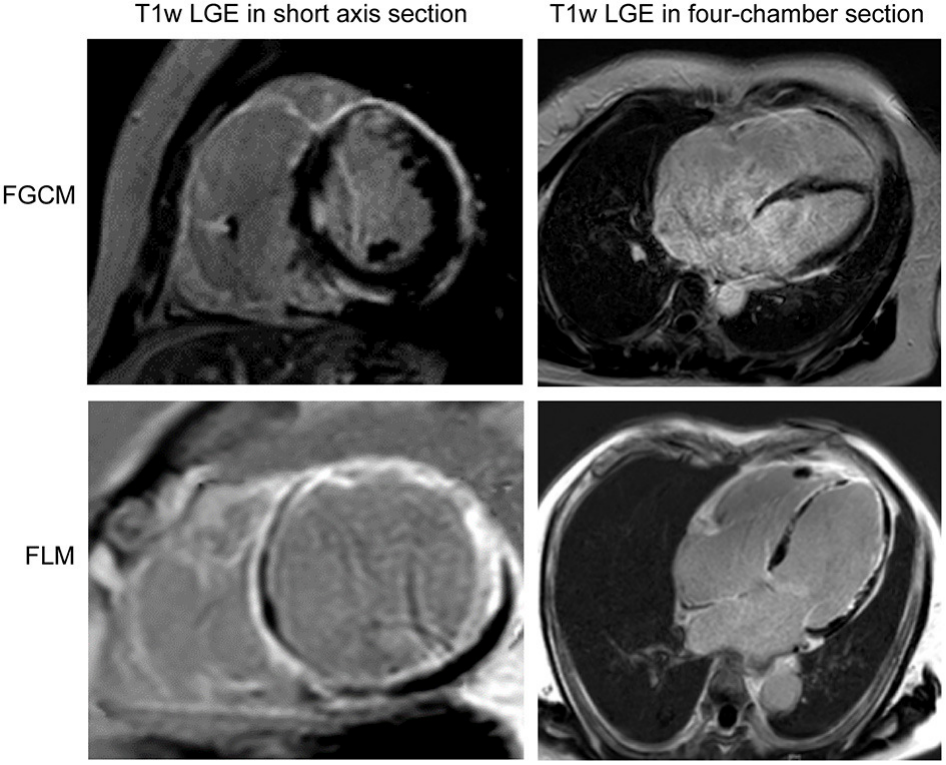
Short axis (left column) and four-chamber section (right column) cardiac magnetic resonance images of representative patients with FGCM (upper line) and FLM (lower line).

The study population consisted of 16 patients, of whom nine were diagnosed with FGCM, while seven were diagnosed with FLM. The representative myocardial histologic appearances and cardiac magnetic resonance images were presented in Figures 1, 2 and Supplementary Figure 1. Several giant cells and widespread lymphocytes, with lesser numbers of neutrophils and eosinophils, could be observed in hematoxylin and eosin (H&E) staining slides. Masson trichrome staining showed the fibrosis of myocardial cells to some extent in the patients with FGCM and FLM. CD3-positive and CD20-positive lymphocytes were observed in both groups, and CD68-positive macrophages were seen solely in FGCM. It seemed that a smaller number of CD8-positive T cells and a larger number of CD4-positive T cells were observed in the FGCM hearts. The cardiac magnetic resonance images showed the widespread distribution of late gadolinium enhancement in patients with FGCM and FLM.

The main characteristics of the study population and a comparison between patients with FGCM and FLM were presented in Table 1. The mean age at disease presentations were 47.9 ± 7.5 and 42.1 ± 12.3 years and the proportion of female patients was 67 and 57% in the FGCM group and FLM group. No significant difference in age and gender was found between the two groups. Arrhythmia symptoms including palpitations, syncope and dizziness were more commonly observed in FGCM patients (56 vs. 29%, *p* = 0.286), but the difference was not statistically significant. There were no statistically significant differences in the baseline New York Heart Association (NYHA) classification and the frequency of life-threatening arrhythmias onset between the two groups.

**Table 1:** Comparison of baseline characteristics, clinical presentation, laboratory examinations, and echocardiography findings in patients with FGCM and FLM.

**Variables**	**Fulminant myocarditis (*****N*** **= 16)**		***P*-value**
	**FGCM (*N* = 9)**	**FLM (*N* = 7)**	
**Demographics**			
Age (years)	47.9 ± 7.5	42.1 ± 12.3	0.267
Female (%)	6 (67)	4 (57)	1.000
BMI (kg/m^2^)	24.2 ± 4.5	22.8 ± 3.9	0.543
Smoke history (%)	3 (33)	0 (0)	0.213
Alcohol history (%)	3 (33)	0 (0)	0.213
**Prodromal symptoms**			
Fever (%)	0 (0)	3 (43)	0.063
GI symptoms (%)	1 (11)	3 (43)	0.262
Respiratory symptoms (%)	2 (22)	1 (14)	1.000
**Primary presenting symptoms**			
Heart failure (%)	3 (33)	4 (57)	0.615
Chest Pain (%)	1 (11)	1 (14)	1.000
Arrhythmia symptoms (%)	5 (56)	2 (29)	0.286
**Vital signs**			
Heart rate (beats/min)	82.2 ± 16.4	88.7 ± 28.8	0.588
Systolic blood pressure (mmHg)	103.7 ± 13.6	95.6 ± 13.7	0.259
Diastolic blood pressure (mmHg)	69.8 ± 9.1	64.0 ± 9.7	0.241
**NYHA functional class**			0.132
II	1 (11)	0 (0)	
III	3 (33)	0 (0)	
IV	5 (56)	7 (100)	
**Life-threatening arrhythmias**			
Advanced atrioventricular block (%)	3 (33)	2 (29)	1.000
MAE (%)	4 (44)	2 (29)	0.633
Cardiac arrest (%)	1 (11)	0 (0)	1.000
**Comorbidity**			
Hypertension (%)	4 (44)	2 (29)	0.633
Diabetes (%)	1 (11)	0 (0)	1.000
**Admission laboratory tests**			
Hb (g/L)	148.0 (132.0–156.0)	132.0 (101.0–150.0)	0.152
WBC (*109/L)	8.1 (6.1–9.0)	13.1 (11.2–20.8)	**0.013**
NEUT (*10^9^/L)	4.8 (4.2–6.6)	11.5 (8.9–17.5)	**0.007**
LYMPH (*109/L)	1.9 (1.3–2.2)	1.5 (0.6–2.0)	0.315
MONO (*109/L)	0.4 (0.3–0.5)	0.8 (0.5–1.6)	**0.030**
PCT (ng/ml)	0.13 (0.11–0.21)	0.81 (0.22–1.42)	0.134
ESR (mm/h)	6.0 (5.0–12.0)	20.5 (7.0–36.0)	0.125
Big-ET (pmol/L)	0.57 (0.55–3.11)	1.56 (1.04–3.30)	0.316
CRP (mg/L)	10.2 (4.1–11.8)	74.1 (29.3–126.8)	**0.003**
Hs-CRP (mg/L)	4.4 (2.0–10.2)	13.6 (12.6–14.6)	**0.004**
CK (IU/L)	45.0 (42.5–68.5)	176.0 (146.0–711.0)	**0.023**
CK-MB (ng/ml)	1.4 (1.0–3.2)	14.6 (3.0–64.9)	**0.025**
LDH (IU/L)	214.0 (193.0–322.5)	1,033.0 (478.0–3,351.0)	**0.001**
CTnI (ng/ml)	0.066 (0.036–0.631)	6.260 (0.153–38.330)	0.085
NT-proBNP (pg/ml)	5,909.5 (4,310.6–11,998.8)	8,831.4 (4,700.7–31,065.2)	0.427
BUN (mmol/l)	6.2 (5.8–9.6)	13.3 (6.4–22.8)	0.064
Cr (umol/l)	98.3 (70.4–122.0)	166.0 (76.5–224.8)	0.223
ALT (IU/L)	38.0 (25.0–61.5)	997.0 (50.0–3,080.0)	**0.030**
AST (IU/L)	37.0 (21.5–43.0)	912.0 (90.0–4,180.0)	**0.001**
PT (s)	14.6 (13.5–16.1)	16.3 (14.7–26.5)	0.090
FT3 (pg/ml)	2.2 (1.9–2.6)	1.6 (1.4–2.1)	**0.017**
FT4 (ng/dl)	1.3 (1.1–1.5)	0.9 (0.8–1.2)	0.057
TSH (uIU/ml)	1.5 (1.3–5.3)	0.31 (0.07–0.58)	**0.004**
**Echocardiography at admission**			
RVEDD (cm)	2.9 ± 0.3	2.4 ± 0.6	**0.034**
LA (cm)	4.1 ± 0.7	3.4 ± 0.9	0.089
LVEF (%)	33.6 ± 16.7	39.3 ± 15.6	0.493
LVEDD (cm)	5.7 ± 1.1	4.6 ± 0.9	0.058
IVS (cm)	0.9 ± 0.2	0.9 ± 0.1	0.694
LVPW (cm)	0.9 ± 0.2	0.9 ± 0.2	0.407
Pericardial effusion (%)	1 (11)	3 (43)	0.262

*The data are expressed as median (interquartile range), mean ± SD, or numbers (percentage). All the statistically significant P-values are indicated in bold*.

*ALT, alanine aminotransferase; AST, aspartate aminotransferase; Big-ET, big-endothelin; BMI, body mass index; BUN, blood urea nitrogen; CK, creatine kinase; CK-MB, creatine kinase isoenzyme; Cr, creatinine; CRP, C-reactive protein; CTnI, cardiac troponin I; ESR, erythrocyte sedimentation rate; FT3, free triiodothyronine; FT4, free thyroxine; GI, gastrointestinal; Hb, hemoglobin; Hs-CRP, high-sensitivity C-reactive protein; IVS, interventricular septal thickness; LA, left atrium; LDH, lactate dehydrogenase; LVEDD, left ventricular end-diastolic diameter; LVEF, left ventricular ejection fraction; LVPW, left ventricular posterior wall; LYMPH, lymphocyte; MAE, malignant arrhythmic events; MONO, monocyte; NEUT, neutrophil; NT-proBNP, N-terminal pro-brain natriuretic peptide; NYHA, New York Heart Association; PCT, procalcitonin; PT, prothrombin time; RVEDD, right ventricular end-diastolic diameter; TSH, thyroid stimulating hormone; WBC, white blood cell*.

*Arrhythmia symptoms: palpitations, syncope, or dizziness. Malignant arrhythmic events: ventricular fibrillation or sustained ventricular tachycardia*.

### Laboratory Findings and Echocardiographic Features

Despite similar demographical and clinical characteristics in those with FGCM vs. those with FLM, there were notable differences in their laboratory and echocardiography findings. The levels of C-reactive protein (CRP) [10.2 (4.1–11.8) mg/L vs. 74.1 (29.3–126.8) mg/L, *P* = 0.003, median with IQR], high-sensitivity C-reactive protein (hs-CRP) [4.4 (2.0–10.2) mg/L vs. 13.6 (12.6–14.6) mg/L, *P* = 0.004], creatine kinase-myoglobin (CK-MB) [1.4 (1.0–3.2) ng/ml vs. 14.6 (3.0–64.9) ng/ml, *P* = 0.025], and aminotransferase (ALT) [38.0 (25.0–61.5) IU/L vs. 997.0 (50.0–3,080.0) IU/L, *P* = 0.030, median with IQR] were significantly lower in patients with FGCM than those with FLM, so was the total number of white blood cells (WBCs) [8.1 (6.1–9.0) ^*^10^9^/L vs. 13.1 (11.2–20.8) ^*^10^9^/L, *P* = 0.013]. For different cell types of peripheral blood WBCs, patients with FGCM exhibited lower neutrophil [4.8 (4.2–6.6) ^*^109/L vs. 11.5 (8.9–17.5) ^*^109/L, *P* = 0.007] and monocyte [0.4 (0.3–0.5) ^*^109/L vs. 0.8 (0.5–1.6) ^*^109/L, *P* = 0.030] counts than FLM. In contrast, the levels of free triiodothyronine (fT3) [2.2 (1.9–2.6) pg/ml vs. 1.6 (1.4–2.1) pg/ml, *P* = 0.017] were significantly higher in patients with FGCM than those with FLM. FGCM patients had greater right ventricular end-diastolic diameter (RVEDD) [2.9 ± 0.3 vs. 2.4 ± 0.6 cm, *P* = 0.034, mean ± SD], and no significant difference in other echocardiographic variables was observed between the two groups.

### Treatments

The treatments of patients with FGCM and FLM during hospitalization are shown in Table 2. A higher proportion of patients with FLM (86 vs. 11%, *p* = 0.009) received methylprednisolone compared to patients with FGCM. In terms of mechanical circulatory support, no significant differences were observed in the use of intra-aortic balloon pump (IABP) (44 vs. 43%, *p* = 1.000) and extracorporeal membrane oxygenation (ECMO) (11 vs. 43%, *p* = 0.262) between the two groups.

**Table 2:** Comparison of treatments during hospitalization in patients with FGCM and FLM.

**Variables**	**Fulminant myocarditis (*****N*** **= 16)**		***P*-value**
	**FGCM (*N* = 9)**	**FLM (*N* = 7)**	
**Drugs**			
Methylprednisolone (%)	1 (11)	6 (86)	**0.009**
Beta-adrenergic blockers (%)	7 (78)	3 (43)	0.302
ACEI/ARB (%)	3 (33)	2 (29)	1.000
MRA (%)	8 (89)	2 (29)	**0.035**
Amiodarone (%)	6 (67)	2 (29)	0.315
Permanent pacemaker (%)	4 (44)	2 (29)	0.633
**Mechanical circulatory support**			
IABP (%)	4 (44)	3 (43)	1.000
ECMO (%)	1 (11)	3 (43)	0.262

*The data are expressed as numbers (percentage). All the statistically significant P-values are indicated in bold*.

*ACEI/ARB, angiotensin converting enzyme inhibitor/angiotensin receptor blockers; ECMO, extracorporeal membrane oxygenation; IABP, intra-aortic balloon pump; MRA, aldosterone receptor antagonist*.

### Survival Analysis

**Figure 3:**
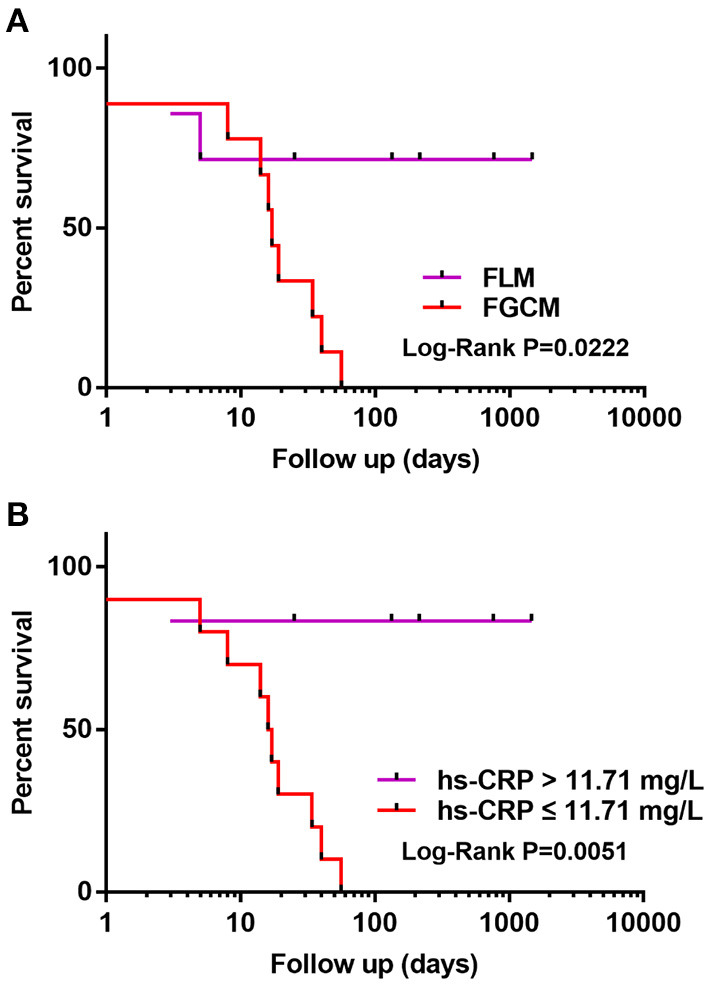
**(A,B)** Kaplan-Meier curve illustrating transplant-free survival in FGCM and FLM patients. Patients with FGCM and lower hs-CRP levels (hs-CRP ≤ 11.71 mg/L) showed a significantly worse prognosis during follow-up of 4 years (log-rank test).

Follow-up data were available for all patients. The mean follow-up period was 175 days (range, 1–1,461 days): 1 patient died, and 10 patients underwent heart transplantation. Figure 3A compares the survival of patients with FGCM vs. FLM. The FGCM group exhibited a significantly lower long-term survival rate compared with FLM group. The 4-year survival rate was 0 in the FGCM group and 71% in the FLM group. Table 3 shows the associations between clinical manifestations, laboratory tests and echocardiography findings, and major clinical events by the Cox univariate and multivariate proportional hazards model. In the univariate Cox analysis, FGCM (hazard ratio = 5.329, 95% confidence interval: 1.102~25.777, *P* = 0.037) and hs-CRP (hazard ratio = 0.882, 95% confidence interval: 0.782~0.994, *P* = 0.039) were independent prognostic factors of FM patients. The Pearson correlation analysis revealed the significant correlation of hs-CRP and fT3 (*r* = 0.745, *P* ≤ 0.001, Supplementary Figure 2). However, the univariate analysis indicated that fT3 (hazard ratio = 2.295, 95% confidence interval: 0.733–7.183, *P* = 0.154) was not an independent prognostic factor. Subsequently, clinically significant factors and variables that were significant (*P* < 0.10) in univariate analysis were included in multivariate analysis, and hs-CRP (hazard ratio = 0.871, 95% confidence interval: 0.761–0.996, *P* = 0.043) remained as an independent prognostic factor of FM patients. Figure 3B demonstrates that the survival rate of patients with lower hs-CRP levels (hs-CRP ≤ 11.71 mg/L) group was low, and the difference between the two groups was statistically significant.

**Table 3:** Univariate and multivariate Cox regression analysis of factors associated with the occurrence of cardiac death and heart transplantation in the overall population.

**Variables**	**Univariate analysis**		**Multivariate analysis**	
	**HR (95% CI)**	***p*-value**	**HR (95% CI)**	***p*-value**
Age	0.990 (0.936–1.046)	0.713	0.982 (0.962–1.058)	0.717
FGCM	5.329 (1.102–25.777)	**0.037**	3.501 (0.359–34.164)	0.281
WBC	0.905 (0.792–1.035)	0.145		
Hs-CRP	0.882 (0.782–0.994)	**0.039**	**0.871 (0.761–0.996)**	**0.043**
CK-MB	0.996 (0.965–1.027)	0.784		
LDH	0.999 (0.998–1.000)	0.171		
ALT	1.000 (0.999–1.000)	0.241		
FT3	2.295 (0.733–7.183)	0.154		
TSH	1.264 (0.941–1.697)	0.120		
LVEF	0.988 (0.944–1.034)	0.603		
RVEDD	3.017 (0.832–10.938)	0.093	1.193 (0.801–1.160)	0.698

*All the statistically significant P-values are indicated in bold*.

*ALT, alanine aminotransferase; CK-MB, creatine kinase isoenzyme; FT3, free triiodothyronine; Hs-CRP, high-sensitivity C-reactive protein; LDH, lactate dehydrogenase; LVEF, left ventricular ejection fraction; RVEDD, right ventricular end-diastolic diameter; TSH, thyroid stimulating hormone; WBC, white blood cell*.

### Differential Diagnosis

**Figure 4:**
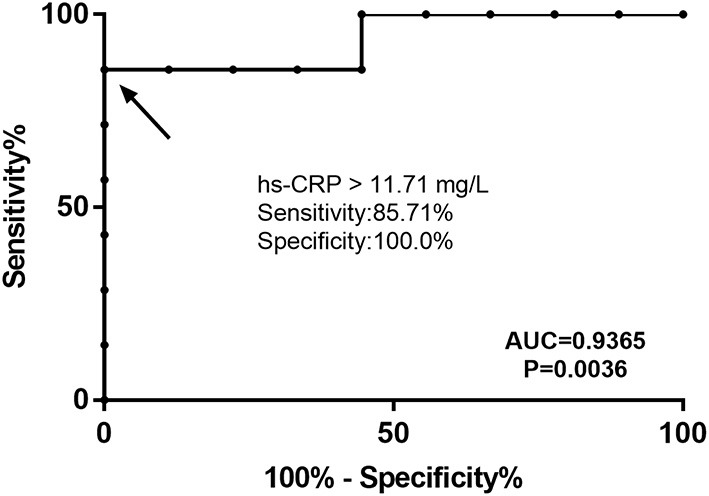
Receiver operating characteristic (ROC) curve for hs-CRP level to distinguish patients with FGCM and FLM. The area under the curve was 0.9365. The best cutoff value for hs-CRP was 11.71 mg/L (sensitivity, 0.8571; specificity, 1.0000).

The receiver operating characteristic (ROC) analysis showed that hs-CRP was a specific and sensitive biomarker that could be used to distinguish FGCM from FLM. The area under the ROC (AUROC) for the levels of hs-CRP in the differential diagnosis between FGCM and FLM was 0.94 (95% CI: 0.4213–0.9964). With an optimal cutoff value of 11.71 mg/L for the levels of hs-CRP, the diagnostic performance for distinguishing between FGCM and FLM were a sensitivity of 85.7% and a specificity of 100.0% (Figure 4).

## Discussion

This study compared the differences between FGCM and FLM and the main findings were: (1) Significant differences in laboratory examinations including inflammatory markers, myocardial enzymes, liver function tests and thyroid hormone were observed between the patients with FGCM and FLM; (2) Patients with FGCM conferred significantly worse prognoses compared to patients with FLM; (3) The level of hs-CRP was of high importance in differential diagnosis and prediction of prognoses for FGCM and FLM.

The results showed that females and middle-aged individuals were more common in patients with FGCM and FLM. Though a lower incidence of prodromal symptoms and heart failure as primary presenting symptoms was found in patients with FGCM, the difference between the two groups was not significant. Therefore, it is difficult to discriminate both disorders solely based on demographic characteristics and clinical manifestations. Previous studies found a rapid progression in patients with FGCM (12, 24). In terms of laboratory findings on admission, we found that the degree of inflammation and myocardial injury in the patients with FGCM were milder than those with FLM. These findings may indicate a more acute clinical course in patients with FLM. The differences in the number and type of peripheral blood WBCs, in conjunction with immunohistochemical findings, might suggest different mechanisms underlying the inflammation response in FGCM and FLM. Rikhi et al. (25) reported that CTLA-4 inhibition resulted in giant cell myocarditis with a predominately CD4+ T cell infiltrate and PD-1 inhibition led to lymphocytic myocarditis with a predominately CD8+ T cell infiltrate. This differential T cell infiltrative might be partly explained by different activation chemokines (26). Ammirati et al. (20) found no difference for the proportion of patients with elevated CRP and myocardial enzymes between the patients with FGCM and FLM. The discrepancies of results between our study and their study may be caused by the variance of the study population, sample size and statistical method. Therefore, more studies are required to draw a specific conclusion in the future.

There is a close relationship between thyroid hormones and cardiac function (27). It was recently noted that lower levels of fT3 were associated with poor prognoses in adult patients with acute myocarditis (28). In this study, there was no known history of previous thyroid disease for all patients. We found that FM patients had significantly lower fT3 levels and patients with FLM showed lower fT3 levels than those with FGCM. Marked activation of inflammatory cytokines including tumor necrosis factor-α (TNF-α) and interleukin (IL)-6 could be involved in the pathogenesis of myocarditis (29, 30). In turn, inflammatory cytokines could decrease serum triiodothyronine levels through regulation of peripheral deiodinase activity (31–34). Virus infection might directly affect thyroid function through extensive injury to the follicular epithelial cells and the parafollicular cells (35). Increased levels of CRP were also found in severe virus infections (36, 37). Furthermore, virus was recognized as one of the most common causes of lymphocytic myocarditis (38, 39). We therefore speculated that lower fT3 levels and higher hs-CRP levels might be relevant to underlying virus infection in patients with FLM. However, laboratory tests of viral infections and thyroid antibodies were lacking in the present cohort, so further study is warranted to explore the mechanisms of thyroid dysfunction in fulminant myocarditis.

In addition, we found that the left ventricular ejection fraction decreased significantly in both FGCM and FLM groups indicating severe myocardial involvement by echocardiography, which conformed with the characteristics of fulminant myocarditis (5). We also found no difference in left ventricular ejection fraction between the two groups and our findings were consistent with those reported in the previous studies (20, 40). A larger right ventricular end-diastolic diameter in patients with FGCM compared to patients with FLM was found in our study. This result suggested that right heart involvement was more frequent in patients with FGCM, and that might be one of the reasons why the patients with FGCM were misdiagnosed as arrhythmogenic right ventricular cardiomyopathy (ARVC).

Supportive measures play a critical role in the management of FM (5). More than half of the patients with FGCM and FLM received mechanical circulatory support (MCS) during hospitalization in this study. MCS improves outcomes by maintaining hemodynamic stability in acute stages, especially for the patients with FLM. Earlier research found that combination immunosuppressive therapy including cyclosporine prolongs median transplant-free survival from 3.0 to 12.4 months for patients with GCM (12). Though we were aware that the application of immunosuppressive therapy could significantly improve the prognoses of GCM (41), a small proportion of patients with FGCM were treated with glucocorticoids in our study. This might be explained by the fact that most of the patients with FGCM were misdiagnosed as other diseases before heart transplantations. It is suggested that the early diagnosis of FGCM is crucial for effective treatments with combination immunosuppressive therapy and improved prognoses.

In agreement with previous studies (20), the prognoses were found to be worse for patients with FGCM than for patients with FLM. Previous studies have identified that hs-CRP level is an independent prognostic predictor in patients with dilated cardiomyopathy and hypertrophic cardiomyopathies (42, 43). Kaneko et al. (44) found that CRP could be a prognostic marker in lymphocytic myocarditis. We found, for the first time, that the level of hs-CRP was considered as an independent predictor of cardiogenic death or heart transplantation in the patients with FM. Moreover, our data showed that hs-CRP level of 11.71 mg/L was an appropriate cutoff point for the differential diagnosis between patients with FGCM and FLM before endomyocardial biopsy or heart transplantation. These findings will be informative for the development of a more precise treatment strategy for patients with FM.

## Limitations

Firstly, our study was conducted within a single medical center and thus single-centered effects cannot be excluded. Secondly, this study had the potential limitations inherent to a retrospective study design which could affect the results. Thirdly, the present study was also limited by a relatively small sample size. Fourthly, the genetic variants in CRP gene that might associated with plasma CRP levels were not examined of patients in our study. Thus, more prospective studies with a large sample size are needed to confirm our findings in the future. Routine testing for genetic variants of CRP is of great significance to clarify the association of these genetic variants with FM.

## Conclusion

In conclusion, our results suggested that the inflammatory response and myocardial damage in the patients with FGCM were milder than those with FLM. Patients with FGCM showed significantly worse long-term prognoses compared to those with FLM. And we identified that hs-CRP could be a useful marker for predicting the prognosis of FM and differential diagnosis between FGCM and FLM.

## Data Availability Statement

The raw data supporting the conclusions of this article will be made available by the authors, without undue reservation.

## Ethics Statement

The studies involving human participants were reviewed and approved by Ethical Committee of Fuwai Hospital. The patients/participants provided their written informed consent to participate in this study.

## Author Contributions

YH designed the whole study, collected the data, and drafted the manuscript. JR, XD, DZ, YQ, CY, YS, JL, FL, WW, HW, and PQ were involved in data analysis, follow-up, and verification. SZ, JH, LY, and HT revised the manuscript critically for important intellectual content. YL approved the final version of the manuscript. All authors contributed to manuscript revision, read, and approved the submitted version.

## Funding

This work was supported by the National Natural Science Foundation of China (81974042), Clinical and Translational Medicine Research Fund of Chinese Academy of Medical Sciences (2019XK320058), Special Research on Basic Resources of Science and Technology (2018FY100606), and National Multidisciplinary Cooperative Diagnosis and Treatment Capacity-Building Project for Major Diseases.

## Conflict of Interest

The authors declare that the research was conducted in the absence of any commercial or financial relationships that could be construed as a potential conflict of interest.

## Publisher's Note

All claims expressed in this article are solely those of the authors and do not necessarily represent those of their affiliated organizations, or those of the publisher, the editors and the reviewers. Any product that may be evaluated in this article, or claim that may be made by its manufacturer, is not guaranteed or endorsed by the publisher.

## References

[1] Gupta S Markham DW Drazner MH Mammen PP. Fulminant myocarditis. Nat Clin Pract Cardiovasc Med. (2008) 5:693–706. 10.1038/ncpcardio1331 18797433

[2] Li S Xu S Li C Ran X Cui G He M. A life support-based comprehensive treatment regimen dramatically lowers the in-hospital mortality of patients with fulminant myocarditis: a multiple center study. Sci China Life Sci. (2019) 62:369–80. 10.1007/s11427-018-9501-9 30850929

[3] Diddle JW Almodovar MC Rajagopal SK Rycus PT Thiagarajan RR. Extracorporeal membrane oxygenation for the support of adults with acute myocarditis. Crit Care Med. (2015) 43:1016–25. 10.1097/CCM.0000000000000920 25738858

[4] Sharma AN Stultz JR Bellamkonda N Amsterdam EA. Fulminant myocarditis: epidemiology, pathogenesis, diagnosis, and management. Am J Cardiol. (2019) 124:1954–60. 10.1016/j.amjcard.2019.09.017 31679645

[5] Veronese G Ammirati E Cipriani M Frigerio M. Fulminant myocarditis: characteristics, treatment, and outcomes. Anatol J Cardiol. (2018) 19:279–86. 10.14744/AnatolJCardiol.2017.8170 29537977 PMC5998855

[6] Ammirati E Veronese G Cipriani M Moroni F Garascia A Brambatti M. Acute and fulminant myocarditis: a pragmatic clinical approach to diagnosis and treatment. Curr Cardiol Rep. (2018) 20:114. 10.1007/s11886-018-1054-z 30259175

[7] Cooper LT Baughman KL Feldman AM Frustaci A Jessup M Kuhl U. The role of endomyocardial biopsy in the management of cardiovascular disease: a scientific statement from the American Heart Association, the American College of Cardiology, and the European Society of Cardiology Endorsed by the Heart Failure Society of America and the Heart Failure Association of the European Society of Cardiology. Eur Heart J. (2007) 28:3076–93. 10.1093/eurheartj/ehm456 17959624

[8] Cooper LT Jr. Giant cell myocarditis: diagnosis and treatment. Herz. (2000) 25:291–8. 10.1007/s000590050023 10904855

[9] Xu J Brooks EG. Giant cell myocarditis: a brief review. Arch Pathol Lab Med. (2016) 140:1429–34. 10.5858/arpa.2016-0068-RS 27922771

[10] Kandolin R Lehtonen J Kupari M. Cardiac sarcoidosis and giant cell myocarditis as causes of atrioventricular block in young and middle-aged adults. Circulation. (2011) 4:303–9. 10.1161/CIRCEP.110.959254 21427276

[11] Davies RA Veinot JP Smith S Struthers C Hendry P Masters R. Giant cell myocarditis: clinical presentation, bridge to transplantation with mechanical circulatory support, and long-term outcome. J Heart Lung Transplant. (2002) 21:674–9. 10.1016/S1053-2498(02)00379-0 12057701

[12] Cooper LT Jr Berry GJ Shabetai R. Idiopathic giant-cell myocarditis–natural history and treatment Multicenter Giant Cell Myocarditis Study Group Investigators. N Engl J Med. (1997) 336:1860–6. 10.1056/NEJM199706263362603 9197214

[13] Hang W Chen C Seubert JM Wang DW. Fulminant myocarditis: a comprehensive review from etiology to treatments and outcomes. Signal Transduction Target Ther. (2020) 5:360. 10.1038/s41392-020-00360-y 33303763 PMC7730152

[14] Ammirati E Cipriani M Lilliu M Sormani P Varrenti M Raineri C. Survival and left ventricular function changes in fulminant vs. non-fulminant acute myocarditis. Circulation. (2017) 136:529–45. 10.1161/CIRCULATIONAHA.117.026386 28576783

[15] Guglin M Nallamshetty L. Myocarditis: diagnosis and treatment. Curr Treat Options Cardiovasc Med. (2012) 14:637–51. 10.1007/s11936-012-0204-7 22927087

[16] Blauwet LA Cooper LT. Myocarditis. Prog Cardiovasc Dis. (2010) 52:274–88. 10.1016/j.pcad.2009.11.006 20109598 PMC5951175

[17] Kilgallen CM Salomon RN Jackson E Surks HK Bankoff MA. Case of giant cell myocarditis and malignant thymoma: a postmortem diagnosis by needle biopsy. Clin Cardiol. (1998) 21:48–51. 10.1002/clc.4960210109 9474466 PMC6655838

[18] Daniels PR Berry GJ Tazelaar HD Cooper LT. Giant cell myocarditis as a manifestation of drug hypersensitivity. Cardiovasc Pathol. (2000) 9:287–91. 10.1016/S1054-8807(00)00049-1 11064276

[19] Ginsberg F Parrillo JE. Fulminant myocarditis. Crit Care Clin. (2013) 29:465–83. 10.1016/j.ccc.2013.03.004 23830649

[20] Ammirati E Veronese G Brambatti M Merlo M Cipriani M Potena L. Fulminant vs. acute nonfulminant myocarditis in patients with left ventricular systolic dysfunction. J Am Coll Cardiol. (2019) 74:299–311. 10.1016/j.jacc.2019.04.063 31319912

[21] Cooper LT Jr ElAmm C. Giant cell myocarditis diagnosis and treatment. Herz. (2012) 37:632–6. 10.1007/s00059-012-3658-1 22930389

[22] Maisch B Ruppert V Pankuweit S. Management of fulminant myocarditis: a diagnosis in search of its etiology but with therapeutic options. Curr Heart Fail Rep. (2014) 11:166–77. 10.1007/s11897-014-0196-6 24723087

[23] McCarthy RE 3rd Boehmer JP Hruban RH Hutchins GM Kasper EK Hare JM. Long-term outcome of fulminant myocarditis as compared with acute (nonfulminant) myocarditis. N Engl J Med. (2000) 342:690–5. 10.1056/NEJM200003093421003 10706898

[24] Okura Y Dec GW Hare JM Kodama M Berry GJ Tazelaar HD. A clinical and histopathologic comparison of cardiac sarcoidosis and idiopathic giant cell myocarditis. J Am Coll Cardiol. (2003) 41:322–9. 10.1016/S0735-1097(02)02715-8 12535829

[25] Rikhi R Karnuta J Hussain M Collier P Funchain P Tang WHW. Immune checkpoint inhibitors mediated lymphocytic and giant cell myocarditis: uncovering etiological mechanisms. Front Cardiovasc Med. (2021) 8:721333. 10.3389/fcvm.2021.721333 34434981 PMC8381278

[26] Oelkrug C Ramage J. Enhancement of T cell recruitment and infiltration into tumours. Clin Exp Immunol. (2014) 178:1–8. 10.1111/cei.12382 24828133 PMC4360188

[27] Klein I Danzi S. Thyroid disease and the heart. Circulation. (2007) 116:1725–35. 10.1161/CIRCULATIONAHA.106.678326 17923583

[28] Zhao Y Wang W Zhang K Tang Y-D. Association between low T3 syndrome and poor prognosis in adult patients with acute myocarditis. Front Endocrinol. (2021) 12:571765. 10.3389/fendo.2021.571765 33763025 PMC7984427

[29] Yamada T Matsumori A Sasayama S. Therapeutic effect of anti-tumor necrosis factor-alpha antibody on the murine model of viral myocarditis induced by encephalomyocarditis virus. Circulation. (1994) 89:846–51. 10.1161/01.CIR.89.2.846 8313574

[30] Matsumori A Yamada T Suzuki H Matoba Y Sasayama S. Increased circulating cytokines in patients with myocarditis and cardiomyopathy. Br Heart J. (1994) 72:561–6. 10.1136/hrt.72.6.561 7857740 PMC1025643

[31] Rozing MP Westendorp RG Maier AB Wijsman CA Frolich M de Craen AJ. Serum triiodothyronine levels and inflammatory cytokine production capacity. Age. (2012) 34:195–201. 10.1007/s11357-011-9220-x 21350816 PMC3260363

[32] Boelen A Kwakkel J Fliers E. Beyond low plasma T3: local thyroid hormone metabolism during inflammation and infection. Endocr Rev. (2011) 32:670–93. 10.1210/er.2011-0007 21791567

[33] Nagaya T Fujieda M Otsuka G Yang JP Okamoto T Seo H. potential role of activated NF-kappa B in the pathogenesis of euthyroid sick syndrome. J Clin Invest. (2000) 106:393–402. 10.1172/JCI7771 10930442 PMC314321

[34] Davies PH Black EG Sheppard MC Franklyn JA. Relation between serum interleukin-6 and thyroid hormone concentrations in 270 hospital in-patients with non-thyroidal illness. Clin Endocrinol. (1996) 44:199–205. 10.1046/j.1365-2265.1996.668489.x 8849575

[35] Wei L Sun S Xu C-h Zhang J Xu Y Zhu H. Pathology of the thyroid in severe acute respiratory syndrome. Hum Pathol. (2007) 38:95–102. 10.1016/j.humpath.2006.06.011 16996569 PMC7112059

[36] Gao R Wang L Bai T Zhang Y Bo H Shu Y. C-reactive protein mediating immunopathological lesions: a potential treatment option for severe influenza A diseases. EBioMedicine. (2017) 22:133–42. 10.1016/j.ebiom.2017.07.010 28734805 PMC5552218

[37] Perez L. Acute phase protein response to viral infection and vaccination. Archiv Biochem Biophys. (2019) 671:196–202. 10.1016/j.abb.2019.07.013 31323216 PMC7094616

[38] Fung G Luo H Qiu Y Yang D McManus B. Myocarditis. Circ Res. (2016) 118:496–514. 10.1161/CIRCRESAHA.115.306573 26846643

[39] Leone O Pieroni M Rapezzi C Olivotto I. The spectrum of myocarditis: from pathology to the clinics. Virchows Arch. (2019) 475:279–301. 10.1007/s00428-019-02615-8 31297595

[40] Davidoff R Palacios I Southern J Fallon JT Newell J Dec GW. Giant cell versus lymphocytic myocarditis. A comparison of their clinical features and long-term outcomes. Circulation. (1991) 83:953–61. 10.1161/01.CIR.83.3.953 1999043

[41] Bang V Ganatra S Shah SP Dani SS Neilan TG Thavendiranathan P. Management of patients with giant cell myocarditis: JACC review topic of the week. J Am Coll Cardiol. (2021) 77:1122–34. 10.1016/j.jacc.2020.11.074 33632487

[42] Zhu L Zou Y Wang Y Luo X Sun K Wang H. Prognostic significance of plasma high-sensitivity C-reactive protein in patients with hypertrophic cardiomyopathy. J Am Heart Assoc. (2017) 6:4529. 10.1161/JAHA.116.004529 28154166 PMC5523755

[43] Ishikawa C Tsutamoto T Fujii M Sakai H Tanaka T Horie M. Prediction of mortality by high-sensitivity C-reactive protein and brain natriuretic peptide in patients with dilated cardiomyopathy. Circ J. (2006) 70:857–63. 10.1253/circj.70.857 16799238

[44] Kaneko K Kanda T Hasegawa A Suzuki T Kobayashi I Nagai R. C-reactive protein as a prognostic marker in lymphocytic myocarditis. Jpn Heart J. (2000) 41:41–7. 10.1536/jhj.41.41 10807528

