# Supplementary material for: Fulminant Giant Cell Myocarditis vs. Lymphocytic Myocarditis: A Comparison of Their Clinical Characteristics, Treatments, and Outcomes
Source: Front Cardiovasc Med. 2021 Dec 3;8:770549. doi: 10.3389/fcvm.2021.770549 (PMC8678080; doi:10.3389/fcvm.2021.770549)
Supplement: Supplementary file 1 [file Data_Sheet_1.docx]

**Supplemental Materials**

**
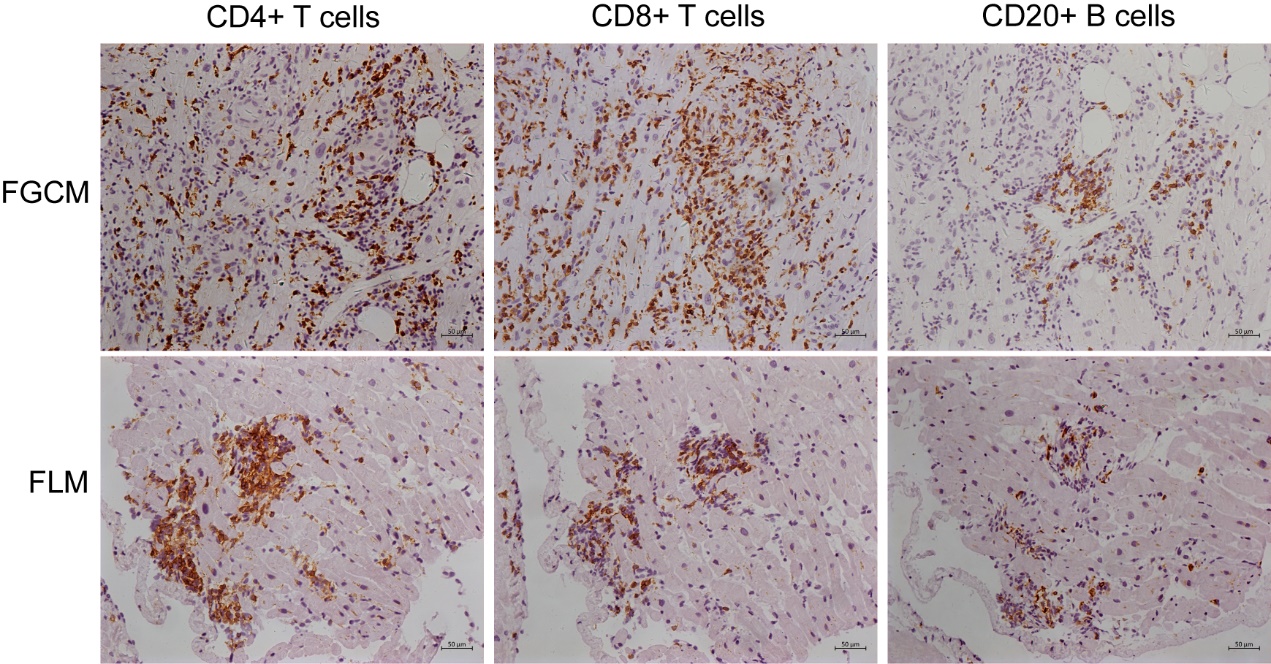
**

**Supplemental Figure 1** Representative immunopathology of patients with FGCM (upper line) and FLM (lower line). left column = staining with anti-CD4 antibody; middle column = staining with anti-CD8 antibody; right column = staining with anti-CD20 antibody.


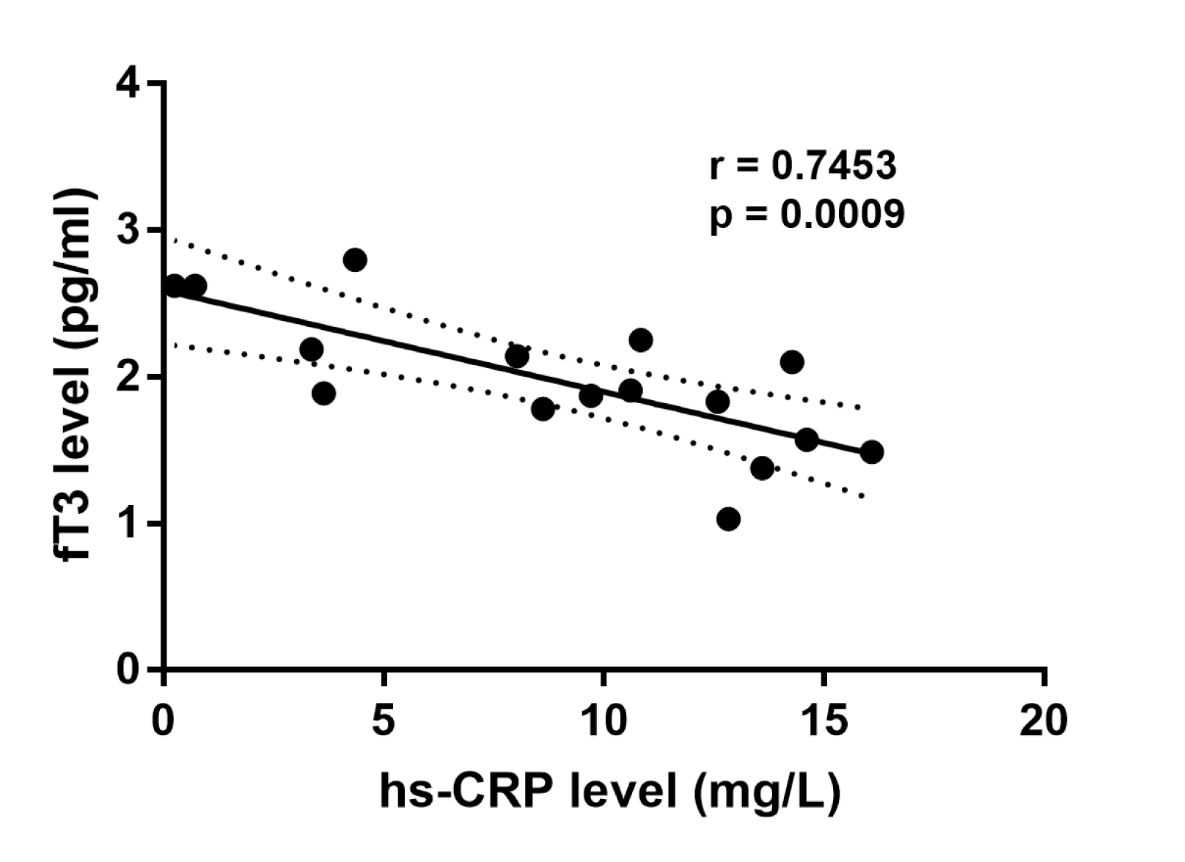


**Supplemental Figure 2** Pearson correlation analysis revealed the significant correlation of levels of hs-CRP and fT3 (r = 0.7453, P ≤ 0.001).
